# Activation of Piezo1 downregulates renin in juxtaglomerular cells and contributes to blood pressure homeostasis

**DOI:** 10.1186/s13578-022-00931-2

**Published:** 2022-12-05

**Authors:** Xiaoqiang Yang, Honghui Zeng, Le Wang, Siweier Luo, Yiming Zhou

**Affiliations:** 1 Guangdong Provincial Key Laboratory of Malignant Tumor Epigenetics and Gene Regulation, Guangdong-Hong Kong Joint Laboratory for RNA Medicine, Sun Yat-Sen Memorial Hospital, Sun Yat-Sen University, Guangzhou, 510120 China; 2 Medical Research Center, Sun Yat-Sen Memorial Hospital, Sun Yat-Sen University, Guangzhou, 510120 Guangdong China

**Keywords:** Piezo1 ion channel, Renin, Calcium signaling, Juxtaglomerular cell, Mechanical stress, Blood pressure homeostasis

## Abstract

**Background:**

The synthesis and secretion of renin in juxtaglomerular (JG) cells are closely regulated by the blood pressure. To date, however, the molecular identity through which JG cells respond to the blood pressure remains unclear.

**Results:**

Here we discovered that Piezo1, a mechanosensitive ion channel, was colocalized with renin in mouse kidney as well as As4.1 cells, a commonly used JG cell line. Activation of Piezo1 by its agonist Yoda1 induced an intracellular calcium increase and downregulated the expression of renin in these cells, while knockout of Piezo1 in JG cells abolished the effect of Yoda1. Meanwhile, mechanical stress using microfluidics also induced an intracellular calcium increase in wildtype but not Piezo1 knockout JG cells. Mechanistically, we demonstrated that activation of Piezo1 upregulated the Ptgs2 expression via the calcineurin-NFAT pathway and increased the production of Ptgs2 downstream molecule PGE_2_ in JG cells. Surprisingly, we discovered that increased PGE_2_ could decreased the renin expression through the PGE_2_ receptor EP1 and EP3, which inhibited the cAMP production in JG cells. In mice, we found that activation of Piezo1 significantly downregulated the renin expression and blood pressure in wildtype but not adeno-associated virus (AAV)-mediated kidney specific Piezo1 knockdown mice.

**Conclusions:**

In summary, these results revealed that activation of Piezo1 could downregulate the renin expression in JG cells and mice, subsequently a reduction of blood pressure, highlighting its therapeutic potential as a drug target of the renin-angiotensin system.

**Supplementary Information:**

The online version contains supplementary material available at 10.1186/s13578-022-00931-2.

## Introduction

The renin-angiotensin system (RAS) plays a crucial role in the maintenance of fluid-electrolyte and blood pressure homeostasis [1, 2]. Renin, the step-limiting protease of the RAS, is produced, stored, and secreted by the renal juxtaglomerular (JG) cells, which are located in the media of the afferent arterioles at the entrance to the glomerulus [3]. According to the previous studies, three important mechanisms trigger the synthesis and secretion of renin: (1) a decrease in blood pressure caused by the reduction in renal perfusion, (2) the detection of a decrease in sodium in the renal tubule by Macula Densa, (3) and beta-1 adrenergic receptor activation via an increase in the activity of the sympathetic system [4–6]. Intriguingly, of these three mechanisms, blood pressure has been suggested to be the most direct and important factor that regulates the RAS. On the one hand, an inverse relationship between renin and blood pressure changes in afferent arteries has been well demonstrated in vivo. On the other hand, an increase in mechanical stress (MS) was shown to inhibit forskolin-induced renin secretion in renin-producing Calu-6 and As4.1 cells as well as primary JG cells [7]. To date, however, the detailed mechanism by which MS regulates the synthesis and secretion of renin remains unclear.

Mechanotransduction, referring to the conversion of MS into electrochemical signals, plays a vital role in a wide variety of physiological and pathophysiological processes in mammalian cells, including touch, proprioception, pain, vascular development, and blood pressure regulation [8–11]. Mechanosensitive ion channels allow the passage of ions in response to increased membrane tension. Coste and colleagues identified the nonselective cation channels Piezo1 and Piezo2 as a novel class of mechanosensitive ion channels [12]. Increasing evidence indicates that Piezo channels are expressed in a wide range of tissues and play important roles in various physiological functions, including vascular tone maintenance, hypertension, bone metabolism, pulmonary vascular remodeling in pulmonary arterial hypertension, macrophage polarization, and stiffness sensing [13–19]. Studies have shown that local blood flow-associated shear stress, in addition to blood pressure-associated cell membrane stretching are key endogenous activators of Piezo channels [14]. Consistently, the renin synthesis and secretion in JG cells is regulated through calcium influx, which is elicited by the increased blood pressure. However, whether Piezo channels are involved in this event remains unclear.

In this study, we demonstrated that the mechanosensitive ion channel Piezo1, but not Piezo2, is functionally expressed in JG cells, and activation of Piezo1 increased intracellular calcium ([Ca^2+^]_i_) level in JG cells, resulting in decreased level of renin synthesis and secretion. Knockout (KO) of Piezo1 in JG cells reduced the effect of Yoda1. In addition, Piezo1-KO significantly reduced the mechanosensation of JG cells. In mice, the activation of Piezo1 significantly decreased renin expression, the systolic blood pressure (SBP) and mean blood pressure (MBP) levels, while specific knockdown of Piezo1 in the kidney abolished these effects of Yoda1. Mechanistically, we demonstrated that Piezo1 activation-induced renin downregulation is mediated via the Ptgs2 (COX-2)-PGE_2_-EP1/3 pathway. In summary, these results suggested that Piezo1 plays an important role in the mechanosensation of JG cells and contributes to blood pressure homeostasis by regulating renin synthesis and secretion, highlighting its therapeutic potential as a drug target of the RAS.

## Results

### Piezo1 is functionally expressed in JG cells

**Fig. 1:**
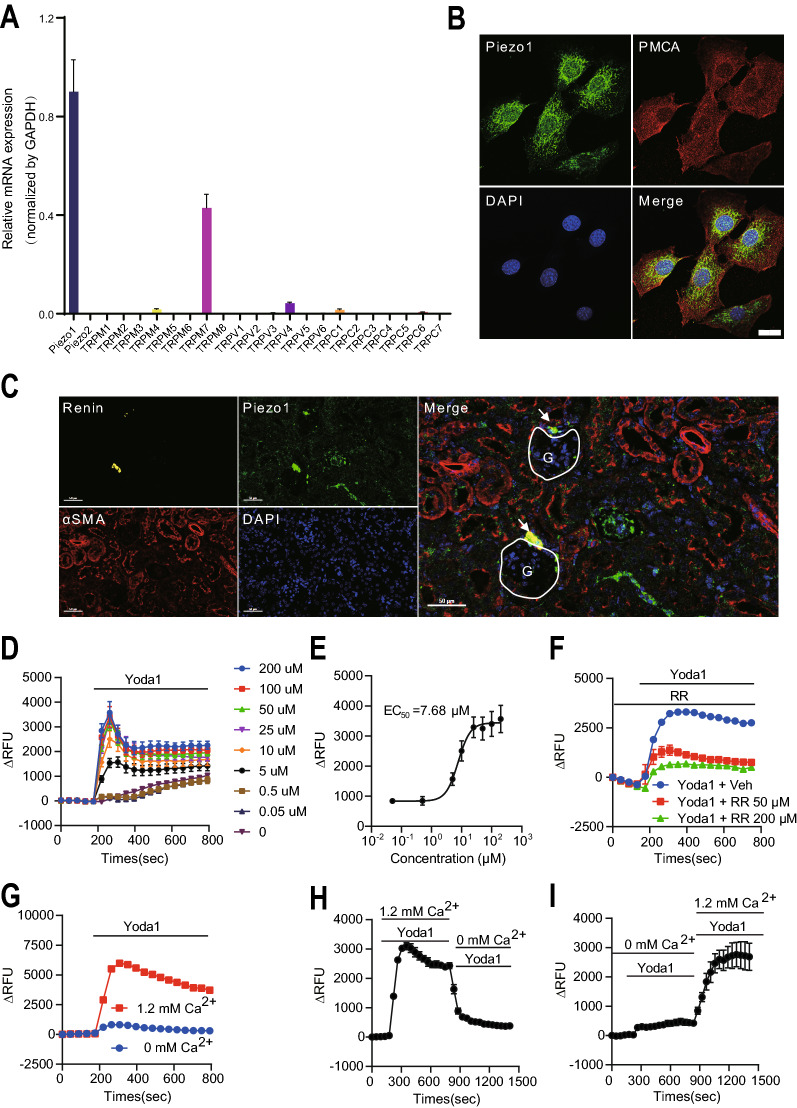
Piezo1 is functionally expressed in JG cells. **A** Screening of the cation channels in As4.1 cells by qRT-PCR. (n = 5). **B** Immunostaining of Piezo1 and Plasma Membrane Calcium ATPase (PMCA, a membrane marker) in As4.1 cells. Scale bar = 20 µm. **C** Multiplexed immunohistochemical staining of Piezo1, renin, and αSMA showing the localization in mouse kidney. Scale bar = 50 µm. White arrows indicate the JG cells; white circles indicate the glomerulus (**G**). **D** and **E** Calcium imaging assay showing the [Ca^2+^]_i_ increase induced by different concentrations of Yoda1 in As4.1 cells. Data are displayed as dose–dependent curves and fitted with Hill equation, indicating the EC_50_ of Yoda1 (n = 4). **F** Calcium imaging assay showing the effect of RR (Ruthenium Red, a cation channel blocker) on Yoda1 induced [Ca^2+^]_i_ increase in As4.1 cells (n = 4). **G** Calcium imaging assay showing the [Ca^2+^]_i_ increase induced by Yoda1 in the presence or absence of extracellular Ca^2+^ in As4.1 cells (n = 4). **H** and **I** Calcium imaging assay showing the [Ca^2+^]_i_ increase induced by Yoda1 while changing the extracellular solution with or without Ca^2+^ in As4.1 cells (n = 4). All data are represented as mean ± SEM

To investigate the expression pattern of ion channels in JG cells, we performed qRT-PCR to detect the mRNA levels of several non-selective cation channels, including Piezo channels, TRPMs, TRPVs and TRPCs in As4.1 cells, a commonly used JG cell line that expresses renin as well as αSMA (Additional file 1: Fig. S1A). The qRT-PCR results showed that Piezo1, but not Piezo2, was highly expressed in As4.1 cells (Fig. 1A). We then verified the subcellular location of Piezo1 in As4.1 cells by immunofluorescence assay. The results showed that Piezo1 was mainly expressed in the membrane and cytoplasm of JG cells (Fig. 1B). To further determine the distribution of Piezo1 in mouse kidneys, we performed the multiplexed immunohistochemical staining of Piezo1 with renin and αSMA in mouse kidneys and found that Piezo1 is highly expressed in JG cells, as well as tubular cells (Fig. 1C, Additional file 1: Fig. S1B).

Next, we investigated the function of Piezo1 in As4.1 cells with a specific agonist Yoda1, using a Fluo-4-based calcium imaging assay. Interestingly, Yoda1 induced a dose-dependent increase in intracellular calcium ([Ca^2+^]_i_) in As4.1 cells, with an EC_50_ of 7.68 µM (Fig. 1D and E). Besides, this increased [Ca^2+^]_i_ level induced by Yoda1 could be blocked by ruthenium red (RR), a blocker of cation channels (Fig. 1F). Moreover, the calcium imaging assay showed that Yoda1 failed to induce a significant [Ca^2+^]_i_ increase in the absence of extracellular calcium (Fig. 1G and H), while adding the calcium to the extracellular solution restored the effect of Yoda1 (Fig. 1I), suggesting that Yoda1 induced the [Ca^2+^]_i_ increase is mainly derived from the extracellular calcium influx but not the release of intracellular bound calcium (such as ER-bound calcium). Taken together, these results demonstrated that Piezo1 is functionally expressed on the plasma membrane of JG cells.

### Activation of Piezo1 reduced renin expression in JG cells

**Fig. 2:**
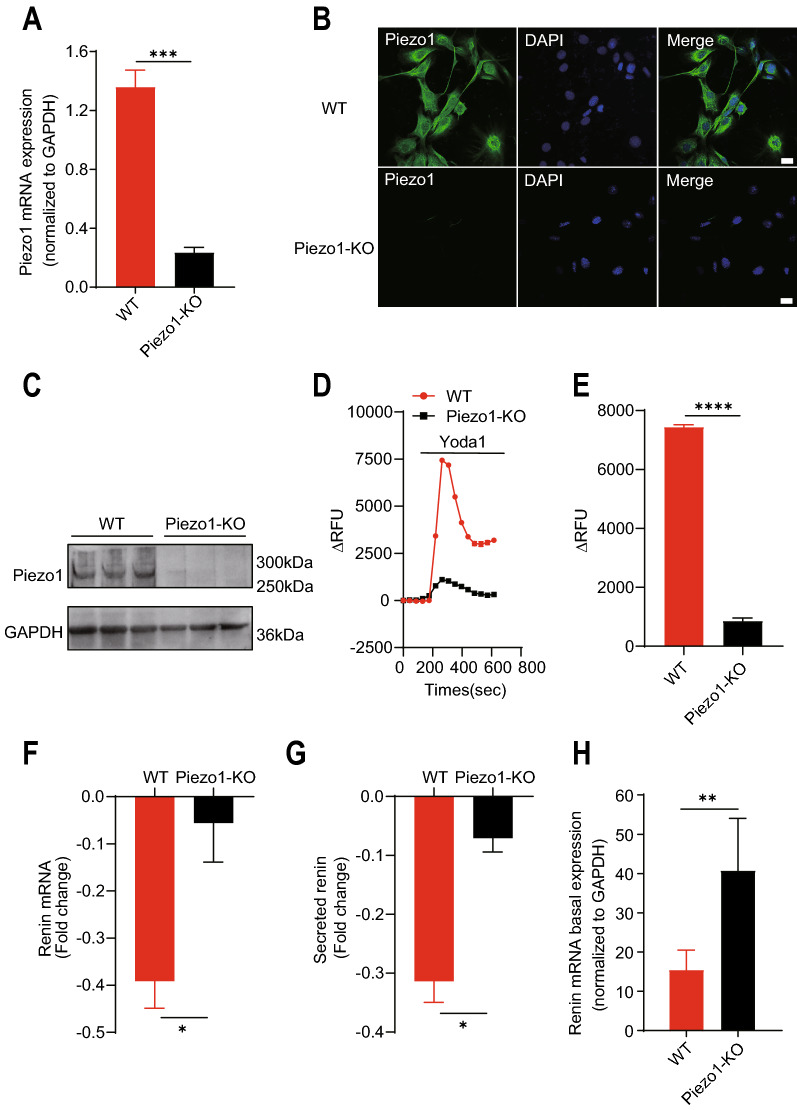
Knockout of Piezo1 abolishes the effect of Yoda1 on renin expression. **A**–**C** Validation of the knockout efficiency of Piezo1 by qRT-PCR (**A**), immunofluorescence (**B**)**,** and Western blotting (n = 3 each) (**C**) in Piezo1-KO As4.1 cells. Scale bar = 20 µm. **D** Calcium imaging assay showing the [Ca^2+^]_i_ increase induced by Yoda1 (20 µM) in WT and Piezo1-KO As4.1 cells (n = 3). **E** Peak values of the [Ca^2+^]_i_ responses induced by Yoda1 from (**D**). **F** and** G** Quantification of the fold changes in mRNA (**F**) and secreted renin **G** levels in WT and Piezo1-KO As4.1 cells after Yoda1 (20 µM) treatment by qRT-PCR and ELISA, respectively (n = 3). **H** Renin mRNA basal expression in WT and Piezo1-KO As4.1 cells. (n = 5). *p < 0.05; **p < 0.01; ***p < 0.001; **** p < 0.0001. Data are represented as mean ± SEM

To investigate the effect of Piezo1 activity on renin synthesis and secretion, we generated a Piezo1-KO As4.1 cell line using the CRISPR/Cas9 system. We confirmed that Piezo1 expression, both at mRNA and protein level, was abrogated in Piezo1-KO As4.1 cells (Fig. 2A–C). In addition, calcium imaging assay results showed that the increase in [Ca^2+^]_i_ induced by Yoda1 was completely abolished in Piezo1-KO As4.1 cells (Fig. 2D and E). Surprisingly, Piezo1-KO not only significantly abolished the effect of Yoda1 on renin expression (Fig. 2F and G), but also exhibited higher renin level at the baseline (Fig. 2H). To further determine the role of Piezo1 in JG cells, we isolated the primary mouse JG cells (Additional file 1: Fig. S2A) and performed the calcium imaging assays and Yoda1 treatment as in As4.1 cells. Results showed that Yoda1 also significantly increased the [Ca^2+^]_i_ level in primary mouse JG cell (Additional file 1: Fig. S2B) and reduced the renin mRNA expression (Additional file 1: Fig. S2C), which is consistent with that in As4.1 cells. Thus, these results together demonstrated that the activation of Piezo1 negatively regulates renin expression in JG cells in a calcium-dependent manner.

**Fig. 3:**
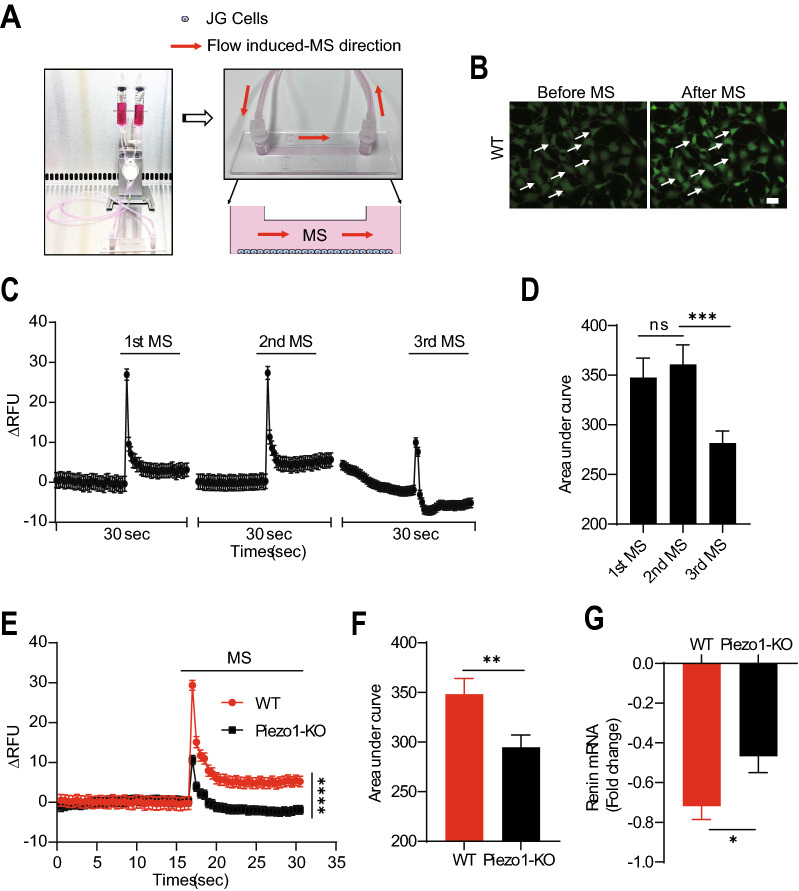
Piezo1 contributes to the mechanosensation in As4.1 cells. **A** Schematic diagram of the flow-induced MS loading system. Red arrows indicate the flow-induced MS direction. **B** Representative images of calcium imaging assay in As4.1 cells before and after MS treatment (30 dyn/cm^2^). White arrows indicate the cells responding to the MS. Scale bar = 20 µm. **C** Representative traces of the [Ca^2+^]_i_ changes in WT As4.1 cells in response to repeated MS (20 dyn/cm^2^, n = 3). **D** Quantification of the AUC of the [Ca^2+^]_i_ increase from (**C**). **E** Representative traces of [Ca^2+^]_i_ changes in WT and Piezo1-KO As4.1 cells in response to the MS (10 dyn/cm^2^, n = 3). **F** Quantification of the AUC of the [Ca^2+^]_i_ increase from (**E**). **G** Quantification of the fold changes in mRNA level in WT and Piezo1-KO As4.1 cells in response to the MS (20 dyn/cm^2^) treatment by qRT-PCR. (n = 3). *p < 0.05; **p < 0.01; ****p < 0.0001. Data are represented as mean ± SEM

Previous studies suggested that MS, such as blood pressure, regulates renin synthesis and secretion. We then hypothesized that Piezo1 contributes to the mechanosensation of JG cells. To test this hypothesis, we applied MS to both WT and Piezo1-KO As4.1 cells using a flow-induced MS loading system (Fig. 3A) and performed the calcium imaging assay. Then real-time dynamic changes of [Ca^2+^]_i_ were observed in As4.1 cells after MS application. The results showed that [Ca^2+^]_i_ was increased immediately after the application of MS in WT As4.1 cells (Fig. 3B). Furthermore, WT As4.1 cells showed a repetitive transient [Ca^2+^]_i_ increase in response to repetitive MS (Fig. 3C and D). Interestingly, Piezo1-KO As4.1 cells exhibited a significantly lower [Ca^2+^]_i_ increase of AUC, peak and plateau values in response to MS (Fig. 3E and F, Additional file 1: Fig. S3A and B). In addition, MS significantly downregulated renin mRNA expression in WT but not in Piezo1-KO As4.1 cells (Fig. 3G, Additional file 1: Fig. S3C), suggesting that Piezo1 plays an important role in mechanosensation and renin expression in JG cells. However, we still observed a reduction in renin expression in Piezo1-KO As4.1 cells, indicating that other channels, such as TRPV4, may also contribute to the mechanosensation in JG cells [20]. Therefore, we also examined the MS to WT and TRPV4-KO As4.1 cells, and found that TRPV4-KO As4.1 cells exhibited a slightly attenuated calcium influx compared with that of Piezo1-KO As4.1 cells in response to MS (Additional file 1: Fig. S3D).

### Piezo1 regulates renin via the calcineurin-Ptgs2 (COX-2)-PGE_2_-EP1/3 pathway

**Fig. 4:**
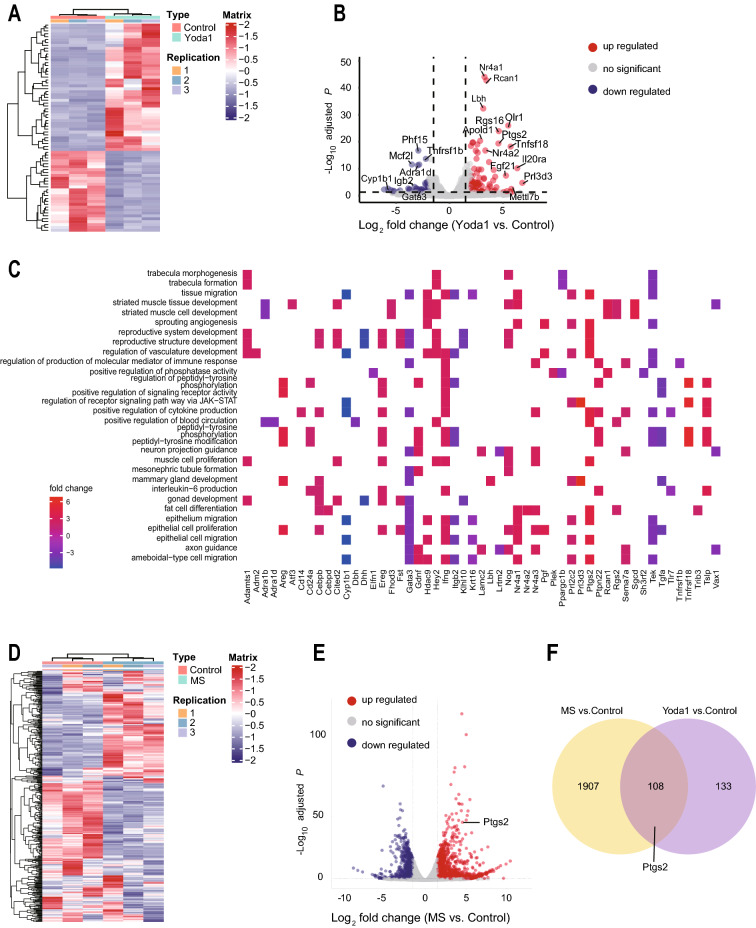
Activation of Piezo1 by Yoda1 and MS induce Ptgs2 expression. **A** and **B** Heatmap and volcano plot of the DEGs from the RNA-seq assay with or without Yoda1 (20 µM) treatment (n = 3). **C** Gene Set Enrichment Analysis of the DEGs. **D** and **E** Heatmap **D** and Volcano plot **E** of the DEGs from the RNA-seq assay with or without MS (20 dyn/cm^2^) in As4.1 cells (n = 3). **F** Venn diagram showing numbers of the overlapped DEGs between Yoda1 and MS groups

To explore the mechanism of how Piezo1 downregulates renin expression, we performed the RNA-seq assay using As4.1 cells with or without Yoda1 treatment. Differentially expressed genes (DEGs) between two groups were shown in a heatmap (Fig. 4A) and volcano plot (Fig. 4B). Gene set enrichment analysis (GSEA) revealed that most of the DEGs are enriched in the vasculature development and regulation of blood circulating cytokine production (Fig. 4C) which indicates the crucial role of Piezo1 in regulation of the blood pressure homeostasis. Next, we validated these DEGs using qRT-PCR in WT and Piezo1-KO As4.1 cells with or without Yoda1 treatment. The qRT-PCR validation results were consistent with most of the DEGs uncovered by the RNA-seq assay (Additional file 1: Fig. S4). Interestingly, we found that the Ptgs2 was highly expressed in JG cells and its expression level was significantly affected by Yoda1 compared with that of other genes. In addition, RNA-seq assay using As4.1 cells with or without MS treatment demonstrated that MS also induced significant changes in Ptgs2 (Fig. 4D and E), which was consistent with the result of Yoda1 treatment (Fig. 4F).

**Fig. 5:**
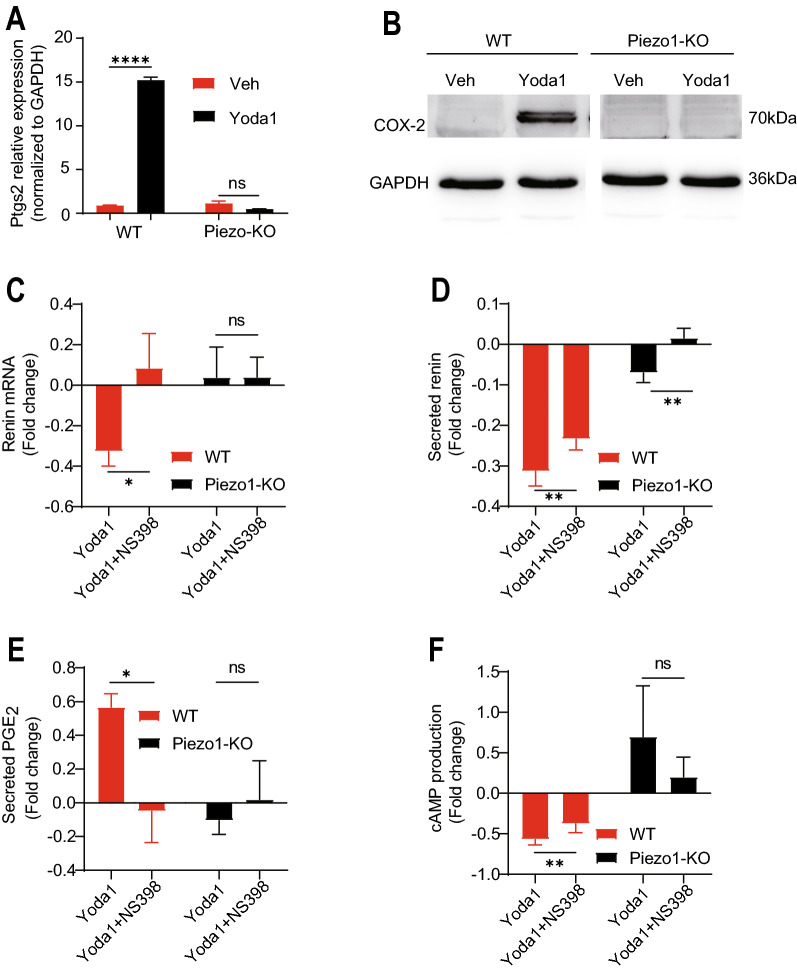
Piezo1 regulates renin expression through Ptgs2 (COX-2). **A** and** B** Quantification of the mRNA and Protein expression level of Ptgs2 (COX-2) in WT and Piezo1-KO As4.1 cells with or without Yoda1 (20 µM) treatment (n = 3). **C** and **D** Quantification of the fold changes in mRNA (**C**) and secreted renin (**D**) levels in WT and Piezo1-KO As4.1 cells with Yoda1 (20 µM) treatment in the presence or absence of NS398 (a COX-2 inhibitor) by qRT-PCR and ELISA, respectively (n = 3). **E** and **F** Quantification of the fold changes of the secreted PGE_2_ and cAMP levels in WT and Piezo1-KO As4.1 cells treated with Yoda1 (20 µM) with or without NS398 (n = 3). *ns* no significant; *p < 0.05; **p < 0.01; ****p < 0.0001. Data are represented as mean ± SEM

Since several reports showed that calcineurin, a downstream molecule of Piezo1, could regulate Ptgs2 expression in the kidney [21, 22]; therefore, we applied two calcineurin inhibitors, FK506 and cyclosporin A (CsA), to As4.1 cells before Yoda1 treatment to investigate whether calcineurin participates in these cells. The results showed that both FK506 and CsA significantly inhibited the expression of Ptgs2 (COX-2) (Additional file 1: Fig. S5A and B), which indicated that Piezo1 regulates Ptgs2 via a calcineurin-dependent pathway in JG cells. Next, changes in Ptgs2 (COX-2) at the mRNA and protein levels were investigated in WT and Piezo1-KO As4.1 cells. Treatment with Yoda1 strongly upregulated the Ptgs2 mRNA as well as its protein COX-2 expression level in WT As4.1 cells (Fig. 5A and B). This effect, however, was completely eliminated in Piezo1-KO As4.1 cells. Previous reports demonstrated that the activation of Ptgs2 (COX-2) affects the renin expression [23, 24]; therefore, we speculated that the activation of Piezo1 reduces the renin expression through upregulation of the Ptgs2 (COX-2) expression. To investigate this hypothesis, NS398, a small-molecule enzymatic inhibitor of COX-2, was applied together with Yoda1 to WT and Piezo1-KO As4.1 cells. Then, we found that NS398 significantly reduced the effects of Yoda1 on renin synthesis and secretion, suggesting that Ptgs2 (COX-2) is an important downstream player of Piezo1 in JG cells (Fig. 5C and D).

Robertson et al. previously reported that COX-2 reduces cAMP levels through the PGE_2_-EP3-Gα_i/o_ pathway [25]. PGE_2_ is a crucial downstream enzymatic product of COX-2 produced by the conversion of arachidonic acid. In the kidney, the COX-2-PGE_2_ cascade is of high importance in regulating fluid metabolism, blood pressure, and renal hemodynamics [26]. In general, PGE_2_ functions by binding four G-protein-coupled receptors designated EP1, EP2, EP3, and EP4 [27, 28]. Therefore, we explored whether Piezo1 regulates renin expression through the COX-2-PGE_2_-EPs pathway in JG cells. Since we found that EP1 and EP3, but not EP2 or EP4, were highly expressed in As4.1 cells using qRT-PCR (Additional file 1: Fig. S5C), we then detected the levels of secreted PGE_2_ and intracellular cAMP in Yoda1-treated WT and Piezo1-KO As4.1 cells. The results showed that Yoda1 significantly increased the secreted PGE_2_ level and decreased intracellular cAMP level in WT but not Piezo1-KO cells (Fig. 5E and F), suggesting that Piezo1 may reduce the cAMP level through increased PGE_2_ secretion in JG cells.

### Activation of Piezo1 reduced blood pressure in mice

**Fig. 6:**
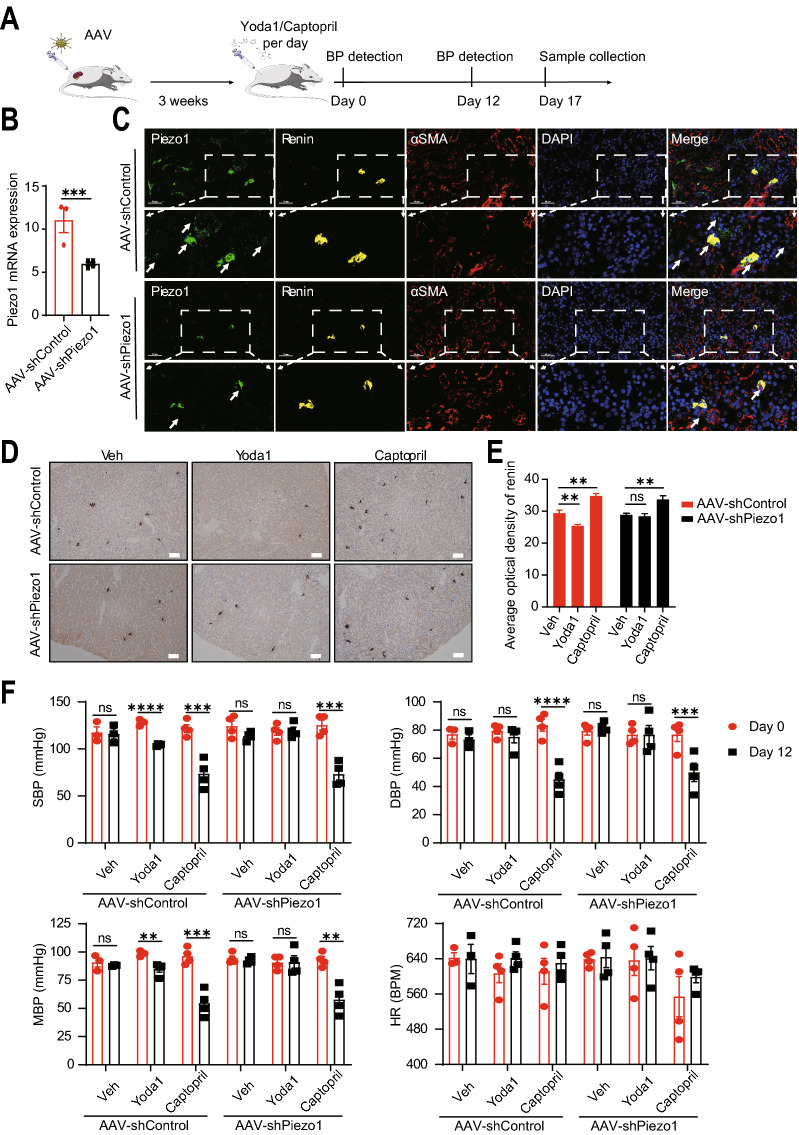
Activation of Piezo1 decreases renin expression and blood pressure in vivo. **A** Schematic design of the animal experiments. **B** qRT-PCR results of the mRNA expression of Piezo1 in kidneys from AAV-shControl (n = 3) and AAV-shPiezo1 (n = 4) mice (37 days post infection). **C** Multiplexed immunohistochemical staining results showing that Piezo1 expression and distribution after AAV infection in mouse kidney. Scale bar = 50 µm. **D** Immunohistochemical staining showing the changes of renin expression in day 12 post Veh/Yoda1/Captopril treatment after AAV infection in mouse kidney (Veh, n = 3, Yoda1, n = 4, Captopril, n = 4). Scale bar = 1000 µm. **E** Quantification of the expression of renin in different treatment groups by the average optical density (AOD) values of IHC. *ns* no significant; ** p < 0.01. Data are represented as mean ± SEM. **F** The levels of systolic blood pressure (SBP), mean blood pressure (MBP), diastolic blood pressure (DBP), and heart rates of mice in day 0 and day 12 post Veh/Yoda1/Captopril treatment, (Veh, n = 3, Yoda1, n = 4, Captopril, n = 4). *ns* no significant; **p < 0.01; ***p < 0.001; ****p < 0.0001. Data are represented as mean ± SEM

To investigate whether Piezo1 activity could regulate renin expression in vivo, we examined the effect of Yoda1 in WT and Piezo1-knockdown mice. A kidney-specific Piezo1-knockdown mouse model was successfully established by a 3-week AAV infection, and Yoda1 was then injected intraperitoneally for 17 days to stimulate Piezo1 (Fig. 6A). Firstly, the knockdown efficiency of Piezo1 was determined by qRT-PCR and multiplexed immunohistochemical staining in mouse kidneys. Results showed that the expression of Piezo1 was reduced in the AAV-shPiezo1 infected mouse kidneys (Fig. 6B and C). Since the in vitro study showed that Yoda1 treatment decreased the renin expression, we then detect the renin expression in mouse kidneys. Interestingly, results showed that Yoda1 treatment significantly reduced the renin expression in AAV-shControl but not AAV-shPiezo1 kidneys (Fig. 6D and E). Furthermore, Yoda1 treatment significantly decreased both SBP and MBP in AAV-shControl but not AAV-shPiezo1 mice (Fig. 6F). These results suggested that the activation of Piezo1 could downregulate renin expression and contribute to the blood pressure homeostasis in vivo.

**Fig. 7:**
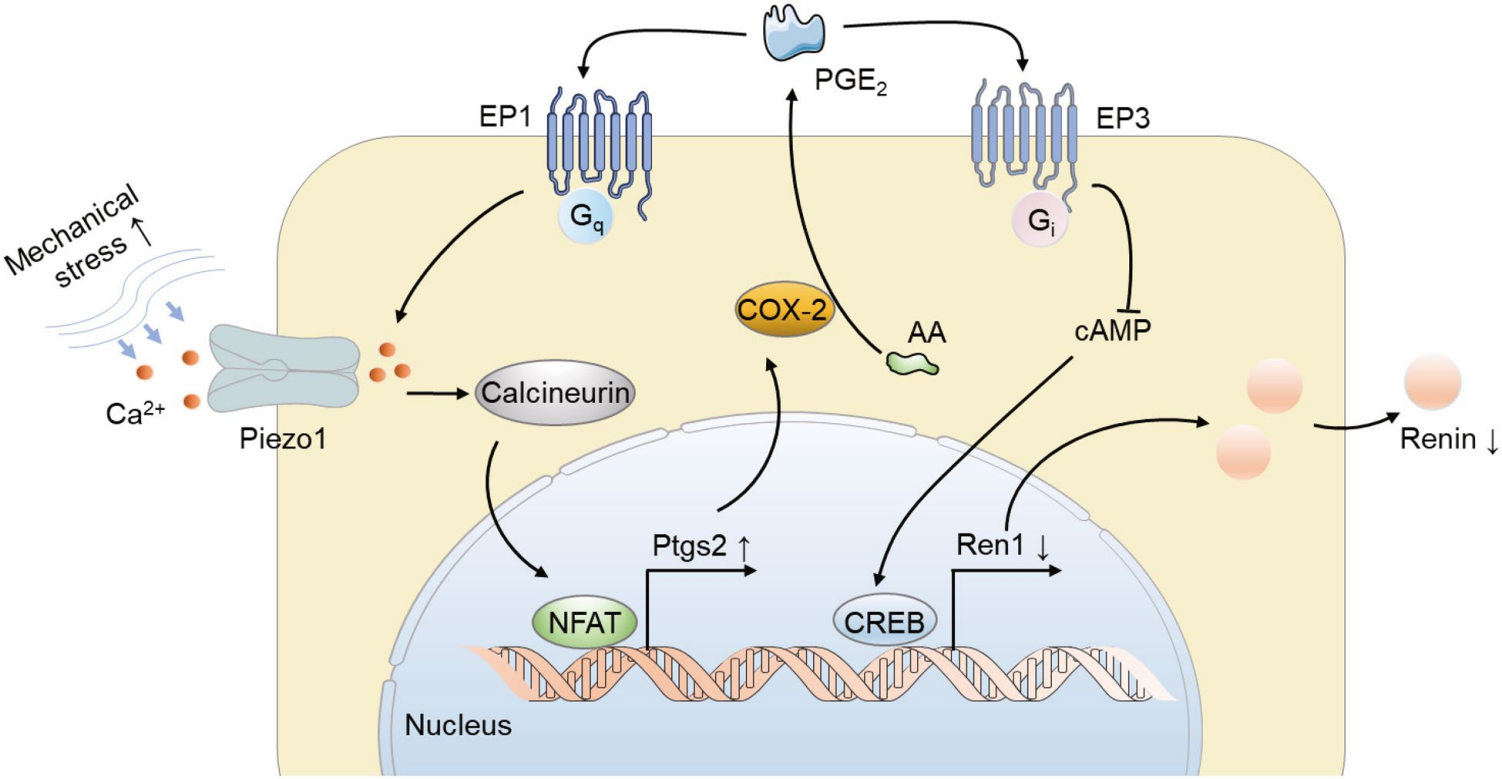
Summary of the mechanism of this study. The increased MS, such as blood pressure, activates the Piezo1 channels and increases the intracellular Ca^2+^ level in JG cells. The increased [Ca^2+^]_i_ induces the Ptgs2 (COX-2) expression via a calcineurin/NFAT pathway and subsequently an increased level of PGE_2_ secretion, which inhibits cAMP production through EP3 and downregulates renin expression

Taken together, our results demonstrated that Piezo1 activation downregulated renin expression through the Ptgs2 (COX-2)-PGE_2_-EP1/3 pathway in JG cells (Fig. 7). Briefly, mechanical stress, such as blood pressure, could activate Piezo1 channel and cause the extracellular calcium influx into the JG cells. The increased intracellular calcium induces the Ptgs2 (COX-2) expression via a calcineurin/NFAT pathway and following the increase of PGE_2_ secretion. The increased PGE_2_, on the one hand inhibits cAMP production through EP3 and downregulates renin expression; on the other hand, elevates the intracellular calcium through EP1, which further reduces renin expression and eventually contributes to blood pressure homeostasis.

## Discussion

The synthesis and secretion of renin from JG cells are crucial for the regulation of the blood pressure in the human body. An inverse relationship between renin production and blood pressure changes in glomerular afferent arterioles has been well documented [29]; however, the detailed mechanism of this process remains unclear. The discovery of Piezo1, a novel mechanosensitive ion channel, led us to wonder whether Piezo1 participates in renin synthesis and secretion from JG cells by sensing the MS generated by the changes of the blood pressure. In this study, we found that Piezo1 is expressed in mammalian JG cells. Both MS and a Piezo1 agonist activated Piezo1 and initiate Piezo1-mediated calcium influx in JG cells in vitro. The activation of Piezo1 resulted in the downregulation of renin expression in JG cells and mice following lowered blood pressure in vivo. We further found that Piezo1 affects renin via the Ptgs2 (COX-2)-PGE_2_-EP1/3 pathway.

In 2010, Coste et al. discovered that two calcium-permeable nonselective cation channels, Piezo1 and Piezo2, are mechanosensitive ion channels that are crucial in mammalian cells in response to various types of MS [12]. Upon MS, including compression, tension, swelling, and shear stress, Piezo channels are activated, inducing calcium influx followed by intracellular signal transmission. Piezo1 is involved in various key biological events, including vascular and lymphatic development [30–32], blood pressure control [19, 33, 34], red blood cell (RBC) volume regulation [35, 36], the perception of force by bladder endothelial cells and renal tubular epithelial cells [37, 38], and bone formation [39, 40]. We screened TRPVs, TRPMs, TRPCs, and Piezos (Piezo1 and Piezo2) expression by qRT-PCR. Among these channels, Piezo1, but not Piezo2, was significantly highly expressed in JG cells. To test whether the Piezo1 channel expressed in As4.1 cells could functionally induce calcium influx, we applied its agonist and antagonist using calcium imaging assay. Intriguingly, on the one hand, we observed a significant rise in [Ca^2+^]_i_ after Yoda1 treatment that could be blocked by RR, a general antagonist for cation channel families. On the other hand, this increase in [Ca^2+^]_i_ in As4.1 cells was observed only in the presence of extracellular calcium after Yoda1 activation, but did not appear when extracellular calcium was removed from the working solutions. As the [Ca^2+^]_i_ increase could be a result of either extracellular calcium influx or [Ca^2+^]_i_ release [41], our results demonstrated that the increased [Ca^2+^]_i_ level in As4.1 cells was due to the extracellular calcium influx.

Previous studies reported the synthesis and secretion of renin is tightly related with the intracellular calcium concentration [42]. Since Yoda1-induced activation of Piezo1 led to calcium influx in As4.1 cells, we wondered whether Piezo1 activation could affect renin expression level. As expected, we observed that renin mRNA as well as protein were significantly reduced after Yoda1 treatment, and this effect was abolished when Piezo1 was knocked out by CRISPR/Cas9. Our results indicate for the first time that Piezo1 plays a key role of regulating renin synthesis and secretion in calcium-dependent manner in As4.1 cells. Piezo1 has been widely reported to be expressed in various tissues and cells and to sense physiological MS in vivo. We wondered whether Piezo1 in As4.1 cells responses to MS and induces [Ca^2+^]_i_ increase. To test this hypothesis, a pump-based MS loading system was used in this study. The results showed that 10–30 dyn/cm^2^ MS could elicit a significant increase in calcium influx in WT As4.1 cells but not Piezo1-KO As4.1 cells, further demonstrating the importance of Piezo1 in the mechanosensation in JG cells. However, we also found that deletion of Piezo1 from As4.1 cells did not completely abolish the MS-induced [Ca^2+^]_i_ increase, suggesting other proteins and structures, including TRPV4, focal adhesions and primary cilia, also likely contribute to mechanosensation in JG cells. Recent studies indicated that TRPV4, an ion channel, is widely expressed in keratinocytes, dorsal root ganglion (DRG) neurons, hippocampal neurons, and urothelial cells, can be activated by mechanical stress [43, 44]. These reports are consistent with our data in TRPV4-KO As4.1 cells, however our results suggested Piezo1 play a more important role in the mechanosensation of JG cells. Moreover, Piezo1 and TRPV4 may interact under some special conditions, as previously reported. For example, Yoneda et al. studied their distinct mechanosensing roles in osteoblastic MC3T3-E1 cells and concluded that TRPV4, but not Piezo1, was sensitive to MS with shear stress upon induction with fluid flow. When both Piezo1 and TRPV4 are highly expressed, the TRPV4-dependent Ca^2+^ response can be induced via activation of Piezo1 [45]. Yarishkin et al. recently reported that Piezo1 mediates fast MS activation and that TRPV4 mediates slow MS activation in human TM cells [30]. Swain et al. reported that Piezo1 acts upstream of TRPV4 to induce pathological changes in endothelial cells due to shear stress [46]. These studies may explain the co-expression of TRPV4 and Piezo1 in As4.1 cells. Possible crosstalk between piezo1 and other sensors and how they sense different kinds of MS, and influence renin in JG cells needs to be further investigated.

Unlike the most secretory cells in human body, renin secretion from the JG cell is inversely related to the [Ca^2+^]_i_ concentrations, which is referred as the “Calcium paradox” [42, 47]. The “Calcium paradox” has been discovered almost forty years, but the detailed mechanism how [Ca^2+^]_i_ regulates renin expression remains unelucidated. Here, we firstly identified that Ptgs2 (COX-2) plays an important role in the renin downregulation induced by Piezo1 activation. Yoda1-induced calcium influx increased the mRNA and protein levels of Ptgs2 (COX-2) in WT but not Piezo1-KO As4.1 cells. Our results showed that Piezo1 activation-induced renin downregulation was abolished by both calcineurin and COX-2 inhibitors. COX-2 is known to increase PGE_2_ production, which is highly important in regulating fluid metabolism, blood pressure, and renal hemodynamics [26]. PGE_2_ functions by binding its four different G-protein-coupled receptors, termed EP1, EP2, EP3, and EP4 [27, 28]. Studies have shown that EP2 and EP4 increase cAMP level, whereas EP3 inhibits cAMP level [27, 28]. EP1 functions via a PLC–dependent calcium signaling pathway [27, 28]. Recent studies reported PGE_2_ inhibits cAMP levels through EP3/G_i/o_ in pancreatic islet cells [25, 48]_._ Our results showed that Yoda1 treatment significantly induced PGE_2_ production and decreased cAMP level in As4.1 cells, while this effect was abolished in Piezo1-KO cells, suggesting Piezo1-Ptgs2 (COX-2)-PGE_2_-EP1/3 pathway play a role in renin expression in JG cells.

Recently, several studies reported that endothelium-specific Piezo1 play a vital role in blood pressure regulation, that activation of Piezo1 in endothelial cell (EC) induced phosphorylation of endothelial NO synthase (eNOS) and regulate blood pressure [18, 34, 49]. Mechanistically, Wang et al. found that endothelial Piezo1 is required for flow-induced ATP release and subsequent P2Y2-Gq/G_11_-mediated activation of downstream signaling, which results in phosphorylation and activation of AKT and endothelial NOS which finally regulates NO formation and blood pressure [34]. Iring et al. identified fluid shear stress–induced Piezo1 activation as a central regulator of endothelial adrenomedullin release and establish the adrenomedullin receptor and subsequent Gs-mediated formation of cAMP as a critical endothelial mechanosignaling pathway regulating basal endothelial NO formation, vascular tone, and blood pressure [18]. Study of Wang et al. demonstrated that COMP (Cartilage Oligomeric Matrix Protein) is a novel Piezo1 regulator that plays a protective role in BP regulation by increasing cellular Ca^2+^ influx, eNOS activity, and nitric oxide production [49]. These studies highlight the importance of Piezo1 in vascular EC for the blood pressure regulation; while our results demonstrated that administration of Piezo1 by Yoda1 could decrease renin expression both in vitro and in vivo, suggesting that there is a direct effect of Piezo1 in JG cells. Since both vascular endothelial cells and JG cells are important players in blood pressure regulation, we believe there is a synergic effect of these cells. In another words, these works together suggest that mammalians may rely on multiple mechanosensitive pathways to maintain blood pressure homeostasis.

In conclusion, our study demonstrated that the mechanosensitive ion channel Piezo1 is functionally expressed in JG cells, and plays a crucial role in renin synthesis and secretion. These findings provide new mechanism of mechanosensation of JG cells. Taken together, these findings suggest that Piezo1 could regulate renin expression, and become as a novel drug target for hypertensive diseases.

## Methods

### Animals

Male C57BL/6J mice at 8-week age were purchased from the Guangdong Medical Laboratory Animal Center (Guangzhou, China). All animals were fed a normal chow diet and water and housed at the Animal Care Facility of the Laboratory Animal Center at Sun Yat-Sen University with a constant temperature of 25 °C and humidity of 60%–65% on a 12-h dark/light cycle under pathogen-free conditions. Animal care and experimental procedures were in accordance with the Guidelines for the Care and Use of Laboratory Animals (NIH Publication, 8th Edition, 2011). Recombinant AAV-mediated Piezo1-knockdown mice were generated by AAV2/9-Piezo1-shRNA virus injection, following a protocol modified from methods described previously [50, 51]. Briefly, the kidneys of all mice were exposed and injected with 10 µL of AAV2/9-Piezo1-shRNA (1.1*10^12^ vg/mL) per site for a total of 7 sites per kidney. AAV2/9-Control-shRNA virus (1.3*10^12^ vg/mL) was administered by the same procedure as a control. The skin was then closed, and the mice recovered for 3 weeks. Then, 20 mg/kg/day Yoda1 and 100 mg/kg/d captopril or an equal volume of saline were intraperitoneally injected into the mice. The blood pressure of the mice was noninvasively measured on days 0 day 12 using tail-cuff plethysmography (BP-2010 series, Softron, Tokyo) as described in a previous study [52]. At 17 days after Yoda1/captopril treatment, the mice were sacrificed, and kidney and blood samples were collected for further experiments. All animal protocols were approved by the Institutional Review Boards of the Animal Care and Use Committees of Sun Yat-Sen University (approval No. SYSU-IACUC-2021-000518).

### Cell culture and calcium imaging assay

The mouse JG cell line As4.1, obtained from the American Type Culture Collection (CRL2193), was maintained in Eagle’s minimal essential medium (ATCC, No. 30-2002) supplemented with 10% FBS, 100 U/mL penicillin, and 100 µg/mL streptomycin at 37 °C in a humidified atmosphere containing 5% CO_2_. As4.1 cells were seeded at 15,000 cells per well and grown overnight to approximately 80–90% confluence. On the second day, the cells were carefully washed 2–3 times with PBS and then quickly but carefully, after which 100 µL of a loading solution containing Fluo-4 dye was added to each well to a final concentration of 5 mM. The plates were incubated at 37 °C for 30 min and then at room temperature for an additional 30 min to achieve stable measurement results. A total of 100 µL of Yoda1 (Piezo1 agonist), GSK1016790A (TRPV4 agonist) or RR at the indicated concentration was added to the cells. All chemical compounds were diluted in HBSS (HyClone, #SH30268.01 with calcium, #30588.01 without calcium), and an equal volume of DMSO in HBSS was used as a control. Then, the fluorescence intensity was measured using instrument settings appropriate for excitation at 494 nm and emission at 516 nm with the Fluo-4 NW Calcium Assay Kit (#F36206).

### RNA-seq experiment

As4.1 cells were treated with DMSO or Yoda1 for 4 h and then washed with PBS twice. Total RNA was extracted with TRIzol, and the concentration of RNA was accurately determined using a Qubit2.0 fluorometer (Thermo Fisher, USA). The integrity of the RNA was detected with an Agilent 2100 bioanalyzer (Agilent, Germany). Quantitative RNA-seq analysis was then performed by Novogene Tech Co. Ltd. (Beijing, China). Data handling and processing were performed on the basis of a previously described bioinformatics pipeline [53].

### Mechanical stress (MS) assay

MS was achieved in a microfluidic chamber (http://ibidi.com). MS is specified in dyn/cm^2^, where 1.0 dyn/cm^2^ is 0.1 Pa or 0.1 N/m^2^. Briefly, 7.5 * 10^5^ Piezo1-KO and WT As4.1 cells were seeded in a µ-Slide I Luer (0.4 mm) fluid chamber slide (Ibidi, Germany) overnight. Before use, the cells were washed with HBSS and then loaded with a Fluo-4 NW working solution following the manufacturer’s instructions. Fluorescence was recorded for 10–20 s before starting fluid flow at the indicated rate for another 3 min. Fluorescent videos were transformed to images by Aoao Video to Picture Converter software, and sixty cells in one of the series images were selected for fluorescence intensity quantification by ImageJ.

### Generation of Piezo1-knockout cell lines

To generate the Piezo1-KO As4.1 cell line, plasmids containing Cas9 and sgRNA targeting Piezo1 (5ʹ-TAGATGCTGCCCCAGCCGTG-3ʹ) were transfected into As4.1 cells via Lipofectamine™ 3000 transfection reagent (#L3000015, Thermo Fisher). Forty-eight hours after transfection, the cells were grown in DMEM supplemented with 10% FBS, 1% penicillin–streptomycin, and puromycin (2 μg/mL; Sigma) for 2 to 3 days. Single-cell clones were sorted into 96-well plates by flow cytometry and then subjected to long-term proliferation. Finally, clones were identified by genome sequencing, qRT-PCR, immunofluorescence analysis, and western blotting to verify the successful knockout of Piezo1.

### Isolation of primary mouse JG cells

Six-week-old C57/BL6 mice were obtained from the Guangdong Medical Laboratory Animal Center (Guangzhou, China). Primary mouse JG cells were isolated as described previously [54]. For one cell preparation, five male C57/BL6 mice that had free access to normal food and water were euthanized by cervical dislocation to avoid adverse effects of anesthesia on the harvested cells. The cells were divided equally into six wells of a six well culture plate (Corning) to a density of 70%. Cells was maintained in Eagle’s minimal essential medium (Hyclone) supplemented with 10% FBS, 100 U/mL penicillin, and 100 µg/mL streptomycin at 37 °C in a humidified atmosphere containing 5% CO_2_. After 72 h, the culture medium was removed and 2 mL fresh prewarmed culture medium was added. Experiments were performed on primary mouse JG cells as designed.

### Quantitative real-time PCR (qRT-PCR)

Total RNA was extracted by using TRIzol reagent (Life Science Technology). Approximately 1000 ng of total RNA was reverse transcribed into cDNA with a reverse transcription PCR Kit (FSQ-101, Toyobo), and qRT-PCR was performed using 2 × Power SYBR Green Master Mix (Applied Biosystems) and a Bio–Rad machine with GAPDH used for the normalization of input cDNA. The qRT-PCR data were analyzed to obtain relative expression. Primer information is listed in Table 1 and Additional file 2: Table S1.

**Table 1:** List of primers used in qRT-PCR

Gene	Species	Forward (5ʹ-3ʹ)	Reverse (5ʹ-3ʹ)
Renin	Mouse	GCACCGCTACCTTTGAACGA	CCACGGGGGAGGTAAGATTG
GAPDH	Mouse	GGAGAGTGTTTCCTCGTCCC	ACTGTGCCGTTGAATTTGCC
Piezo1	Mouse	GTCATGGACTGGGTGTGGAC	TGGGCTGGGGGTATTTCTTC
Piezo2	Mouse	ACTCGTCTGCATCCTACACC	ATTGACTTTGGCATGGCTGT
TRPM1	Mouse	CCTGGCCTGAAGGTCATCAT	CTGCATTTGCATCCACATTCTCT
TRPM2	Mouse	AAGCCTCTCGAAGCCAAGAC	CAGATCTGCAGGCTTCCACT
TRPM3	Mouse	CGAGGACAGCGCAGAGAG	CAGGATTTCTGAGCCGGTGT
TRPM4	Mouse	CTTTGGGGCAGCCGTAGTT	TCAGACAGCCGGAGAAAGTTG
TRPM5	mouse	ATCACGAGCAACAGCCCTGAG	TTGCATGGTGGCTTTCCAGTC
TRPM6	Mouse	TCACCACCATTATTCAGCCATTG	GATGGTCACTTCCTCTCCTGTG
TRPM7	Mouse	GCAGCTTTGTTACCGGATTGG	ACGGGCTTAAATGGAGAAGCA
TRPM8	Mouse	GGTCATTTGGGAGCAGACCA	TTGGCCAGTTCCTCCGATTC
TRPV1	Mouse	AGCGAGTTCAAAGACCCAGAG	TCTGTCTTCCGGGCAATGTC
TRPV2	Mouse	TGGGCGTCAGTGTTTTAGGG	GAGGAAGTTCTGGGGTGGTG
TRPV3	Mouse	GATTTGGAGTAGCGCTGGC	CAGGCCTATGGTGAGCTTGA
TRPV4	Mouse	CATTAACGAGGACCCTGGCA	CCGAGGACCAACGATCCCTA
TRPV5	Mouse	GCAAGAAGACATGGGGGCTA	AGTGGAGACTCCCAAATACTTTT
TRPV6	Mouse	CTGGAGAGCACAGGTTGTGG	CCAAGACCATACTCTCGCCC
TRPC1	Mouse	CGTGCGACAAGGGTGACTAT	CTCCCAAGCACATCTACGCA
TRPC2	Mouse	GGACCCCCTTTCGCCACA	CGAGGCATCGGAACTGGATTC
TRPC3	Mouse	TGGGTAACTCAAAGTCCAGGTTAAA	TGAGAATGCTGTTAAAACTGTGTGA
TRPC4	Mouse	GAAGAGCCAGAGCGAAGAAGAA	ACCCCGTGAAGCTAATCCTTT
TRPC5	Mouse	GGCACCTTACCACCTCCTTT	AGCATGACGTTCTGTGAAGC
TRPC6	Mouse	CTCTGGTTTACGGCAGCAGA	CGCAATATCTTCCCCATCTTGC
TRPC7	Mouse	CTCTCAGGCTTACGGCAACA	AGGGTTTGTCCTAGCTTGCTG
Ptgs2	Mouse	GCTCAGCCAGGCAGCAAATC	ACCATAGAATCCAGTCCGGGT

### Western blotting

Cells were washed three times with PBS and lysed on ice in RIPA buffer containing 1% protease inhibitor cocktail (FuDe Biotech). The protein concentration was determined with a BCA protein assay kit (Beyotime). Proteins were electrophoresed on a 10% SDS–PAGE gel and transferred to a PVDF membrane (Millipore). Then, the membrane was incubated overnight at 4 °C with anti-Piezo1 (#15939-1-AP, Proteintech), anti-COX-2 (#12375-1-AP, Proteintech), or anti-GAPDH (#2118S, CST). Proteins were detected by electrochemiluminescence assay (WBKLS0100, Millipore). Full range gels of western blots are shown in Additional file 1: Fig. S6.

### Immunofluorescence assay

Cells were gently washed twice with PBS, fixed with 4% PFA for 15 min, washed with PBS to remove residual PFA, and then treated with 0.1% Triton X-100 for 5 min following washing with PBS wash three times. Cells were blocked with 5% bovine serum albumin (BSA) for 1 h at room temperature and incubated with the following different primary antibodies overnight at 4 °C: anti-Renin (1:100 dilution, #ab212197, Abcam), anti-Piezo1 (1:100 dilution, #APC-087-25, Alomone), anti-PMCA (1:100 dilution, #MA3-914, Sigma-Aldrich), and anti-αSMA (1:200 dilution, #ab5694, Abcam). Samples were washed with 0.1% PBST buffer (PBS + 0.1% Tween 20) three times for 10 min each and incubated with fluorescently conjugated goat anti-rabbit or goat anti-mouse IgG secondary antibody (Thermo Fisher) for 2 h at room temperature before being washed with 0.1% PBST buffer another three times. The nuclei were counterstained with 4′6′-diamidino-2 phenylindole (DAPI) at room temperature for 5 min, and the cells were finally washed with PBST three times. Fluorescent images were captured using an Olympus IX73 inverted fluorescence microscope or a Zeiss LSM 710 confocal microscope.

### Immunohistochemical staining

The immunohistochemical analysis was performed on paraffin-embedded tissues as previously described [55], Briefly, the deparaffined sections were incubated with 0.3% H_2_O_2_ in methanol to inhibit endogenous peroxidase activity, and non-specific binding was blocked by incubating sections with 5% BSA for 1 h at room temperature. The sections were probed with antibodies against renin (1:100 dilution, #ab212197, Abcam), except they were counterstained with hematoxylin if applicable. The stained sections of each group were examined of the average optical density (AOD) of renin protein. Five randomly fields of each section were used for analyzing the positive staining as previously reported [55, 56] using the ImageJ software, and the AOD values of each sample were used as an index of the expression of renin.

For multiplexed immunohistochemical staining using the Opal protocol [57, 58], briefly, the slides were deparaffinized in xylene and rehydrated in ethanol. Antigen retrieval was performed in citrate buffer (pH 6.0) using microwave heating (MWT). Primary rabbit antibodies for renin (1:100 dilution, #ab212197, Abcam) were incubated for 1 h in a humidified chamber at room temperature, followed by detection using the rabbit SuperPicture Polymer Detection HRP kit. Visualization of renin was accomplished using 1 × Opal 570 TSA Plus, after which the slide was placed in citrate buffer (pH 6.0) and heated using MWT. In a serial fashion, the slide was then incubated with primary rabbit antibodies for piezo1 (1:100 dilution, #15939-1-AP, Proteintech). Samples for 1 h in a humidified chamber at room temperature, followed by detection using the rabbit SuperPicture Polymer Detection HRP kit. Piezo1 was visualized using 1 × Opal 620 TSA Plus. The slide was again placed in citrate buffer (pH 6.0) and subject to MWT, and then incubated with primary rabbit antibodies for αSMA (1:100 dilution, #ab5694, Abcam) for 1 h in a humidified chamber at room temperature, followed by detection using the rabbit SuperPicture Polymer Detection HRP kit. αSMA was then visualized using 1 × Opal 690 TSA. The slide was again placed in citrate buffer (pH 6.0) and heated using MWT. Nuclei were subsequently visualized with DAPI (1:2000) for 5 min, and the section was coverslipped using Vectashield Hardset mounting media. Using this Opal method, three primary antibodies were sequentially applied to a single mouse kidney slide. Visualization of 4-color Opal slides can be performed using Mantra or Vectra Quantitative Pathology Imaging Systems.

### ELISA

The cell supernatants were harvested and centrifuged for 10 min at 10,000 rpm to remove the cell debris and then used to determine the concentrations of PGE_2_ (#CEA538Ge, Cloud-Clone) and renin (#ab193728, Abcam) with ELISA test kits following the manufacturer's instructions. The absorbance at 450 nm was determined with a microplate reader (Quant, BioTek). To determine cAMP concentrations, treated cells were lysed with 0.1 M HCl and analyzed with a kit (#ab133051, Abcam) following the manufacturer’s instructions.

### Statistics analysis

All experiments were conducted at least three times in an independent manner, and all results are shown as the mean ± SEM. All statistical analyses were performed by GraphPad Prism v9.0 using Student’s t test if not specifically noted. Statistical significance was indicated if P < 0.05.

## Supplementary Information

**Additional file 1: Fig. S1.** Detection of renin in JG cells. (A) Immunofluorescence results showing the renin and αSMA expression in As4.1 cells; PMCA, a plasma membrane marker. Scale bar = 20 µm. White arrow: renin signal. (B) Immunofluorescence results showing the Piezo1 and αSMA co-expressed in mouse kidney. Scale bar = 50 µm. Green arrows: Piezo1 signals; white arrows: arterials stained by αSMA; white circles: glomerulus (G). **Fig. S2.** Activation of Piezo1 decreases renin expression in primary JG cells. (A) Immunofluorescence results showing the αSMA and renin co-expressed in primary mouse JG cells. Scale bar = 100 µm. (B) Calcium imaging assay showing the [Ca^2+^]_i_ changes in the presence or absence of Yoda1 (20 μM) in primary mouse JG cells (n = 4). (C) Quantification of renin mRNA expression by qRT-PCR in the presence or absence of Yoda1 (20 μM) in primary mouse JG cells (n = 3). *** p < 0.001. Data are represented as mean ± SEM.** Fig. S3.** Mechanosensation of Piezo1-KO As4.1 cells. (A and B) Calcium imaging assay showing the Peak (A) and plateau (B) values of the [Ca^2+^]_i_ responses induced by MS. (n = 3) (C) Quantification of the mRNA level in WT and Piezo1-KO As4.1 cells in response to the MS (20 dyn/cm^2^, n = 3). (D) Calcium imaging assay showing the [Ca^2+^]_i_ changes in WT As4.1 cells in the presence or absence of extracellular Ca^2+^ after the MS (30 dyn/cm^2^, n = 3) treatment; the [Ca^2+^]_i_ changes in Piezo1-KO as well as in TRPV4-KO As4.1 cells in the presence of extracellular Ca^2+^ after the MS (30 dyn/cm^2^, n = 3). ns, no significant; * p < 0.05; **** p < 0.0001. Data are represented as mean ± SEM. **Fig. S4.** qRT-PCR validation of DEGs in WT and Piezo1-KO As4.1 cells. DEGs from RNA-seq analysis were validated by qRT-PCR in WT and Piezo1-KO As4.1 cells with or without Yoda1 (20 µM) treatment. Red and blue colors of graph titles indicate the upregulated and downregulated genes, respectively (n = 3). ns, no significant; * p < 0.05; ** p < 0.01; *** p < 0.001; **** p < 0.0001. Data are represented as mean ± SEM. **Fig. S5.** Inhibition of calcineurin reduces the Ptgs2 (COX-2) expression. (A and B) Quantification of the expression levels of the Ptgs2 (COX-2) mRNA (A) and protein (B) expression with or without FK506 (20 µM) and CsA (Cyclosporin A, 10 nM) treatments (n = 3) in As4.1 cells for 10 hours. (C) Quantification of the expression levels of PGE_2_ receptors by qRT-PCR in WT and Piezo1-KO As4.1 cells (n = 3). ns, no significant; * p < 0.05; **** p < 0.0001. Data are represented as mean ± SEM. **Fig. S6.** Full range gels of Western blotting. Red boxes indicate the cropped regions shown in the corresponding figures.

**Additional file 2: Table S1.** List of primers used in qRT-PCR in supplementary figures.

## Data Availability

The datasets used and/or analyzed during the current study are available from the corresponding author on reasonable request.

## Abbreviations

JG: Juxtaglomerular
AAV: Adeno-associated virus
RAS: Renin-angiotensin system
MS: Mechanical stress
KO: Knockout
BP: Blood pressure
SBP: Systolic blood pressure
MBP: Mean blood pressure
DBP: Diastolic blood pressure
DEGs: Differentially expressed genes
GSEA: Gene set enrichment analysis
CsA: Cyclosporin A
