# Supplementary material for: Activation of Piezo1 downregulates renin in juxtaglomerular cells and contributes to blood pressure homeostasis
Source: Cell Biosci. 2022 Dec 5;12:197. doi: 10.1186/s13578-022-00931-2 (PMC9720979; doi:10.1186/s13578-022-00931-2)

Fig. S1

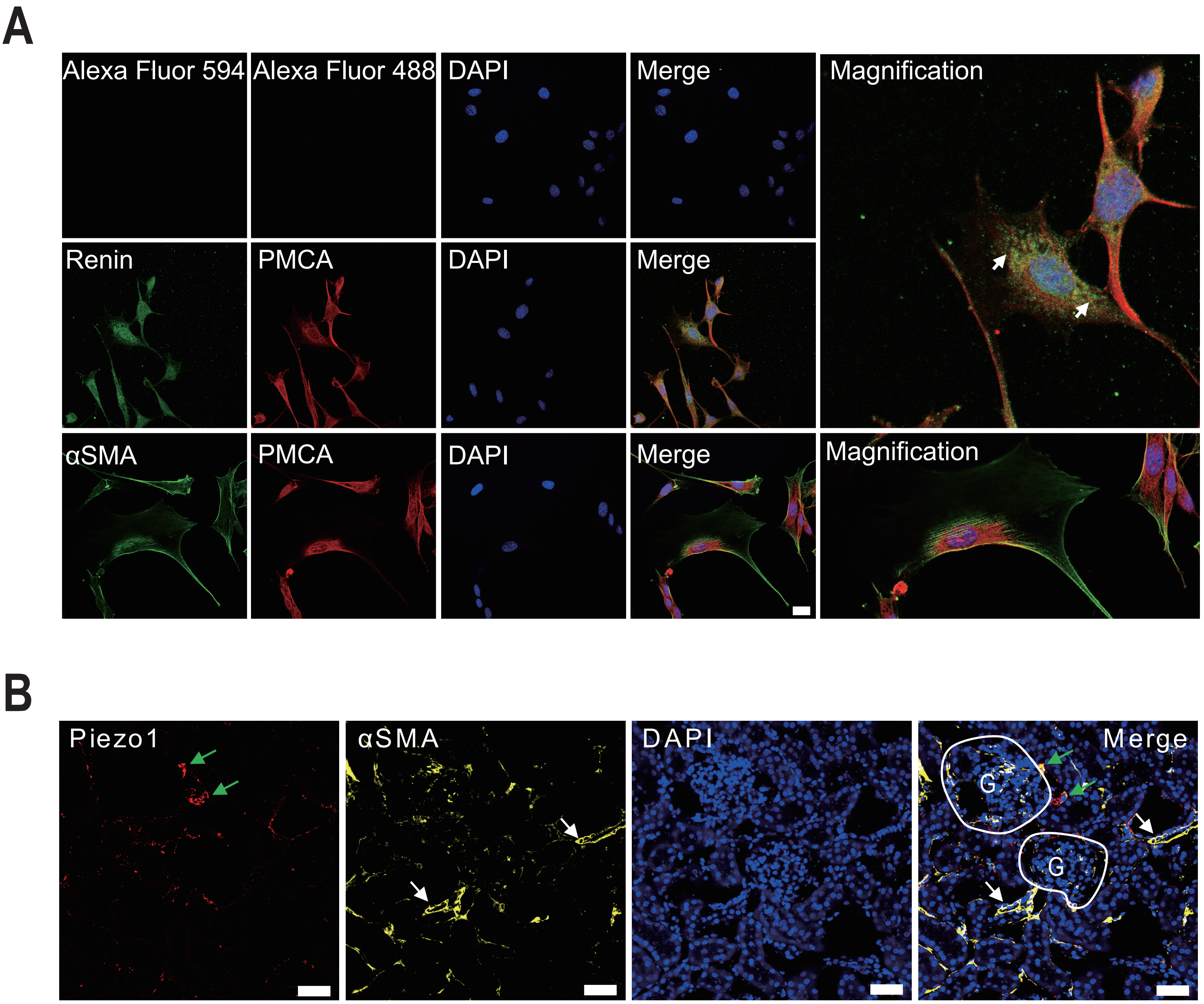
A
Alexa Fluor 594
Alexa Fluor 488
DAPI
Merge
Magnification
Renin
PMCA
DAPI
Merge
αSMA
PMCA
DAPI
Merge
Magnification
B
Piezo1
αSMA
DAPI
Merge
G
G

Fig. S2

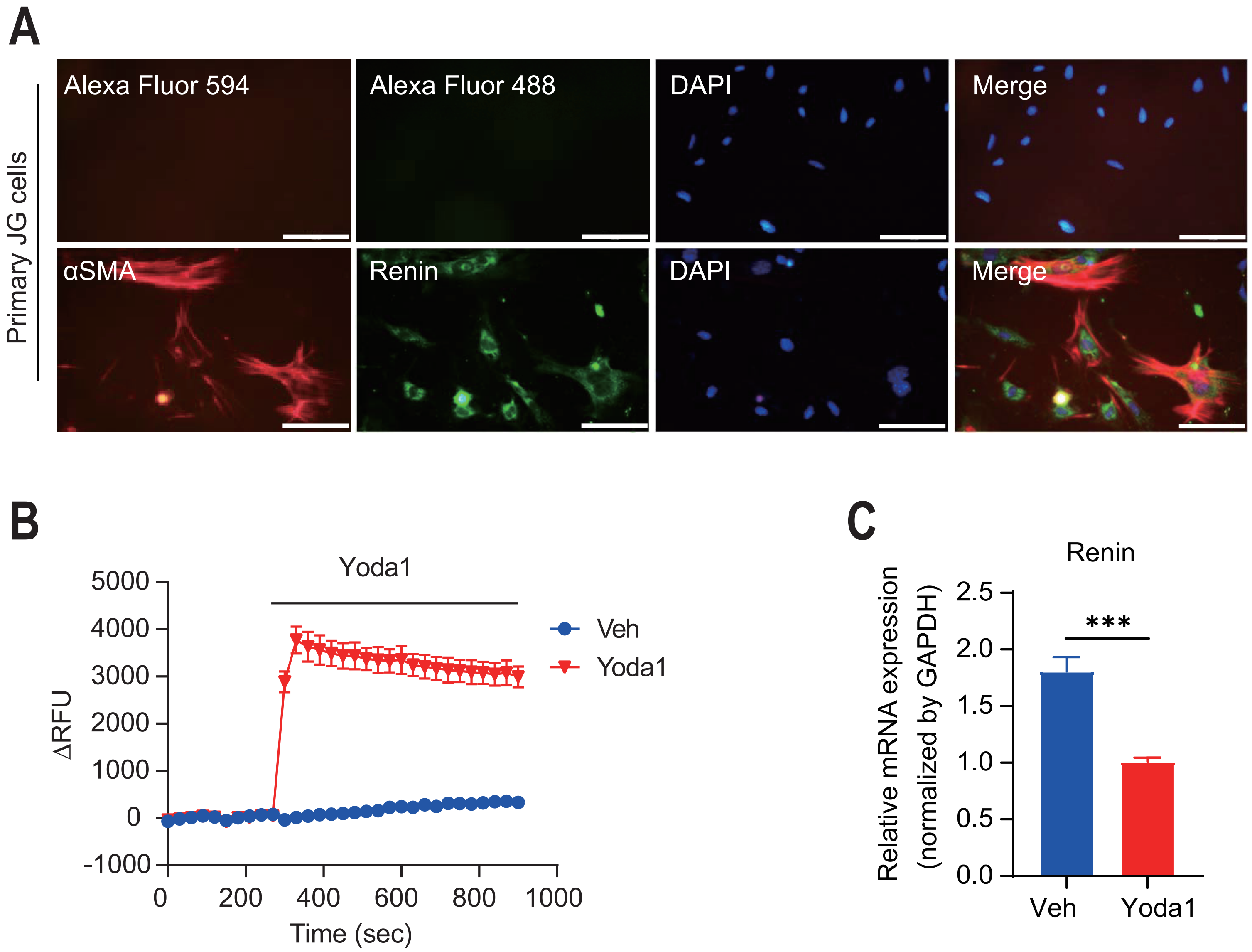
A
Primary JG cells
Alexa Fluor 594
Alexa Fluor 488
DAPI
Merge
αSMA
Renin
DAPI
Merge
B
Yoda1
5000
4000
3000
2000
1000
0
-1000
ΔRFU
0
200
400
600
800
1000
Time (sec)
Veh
Yoda1
C
Renin
Relative mRNA expression
(normalized by GAPDH)
2.5
2.0
1.5
1.0
0.5
0.0
***
Veh
Yoda1

Fig. S3

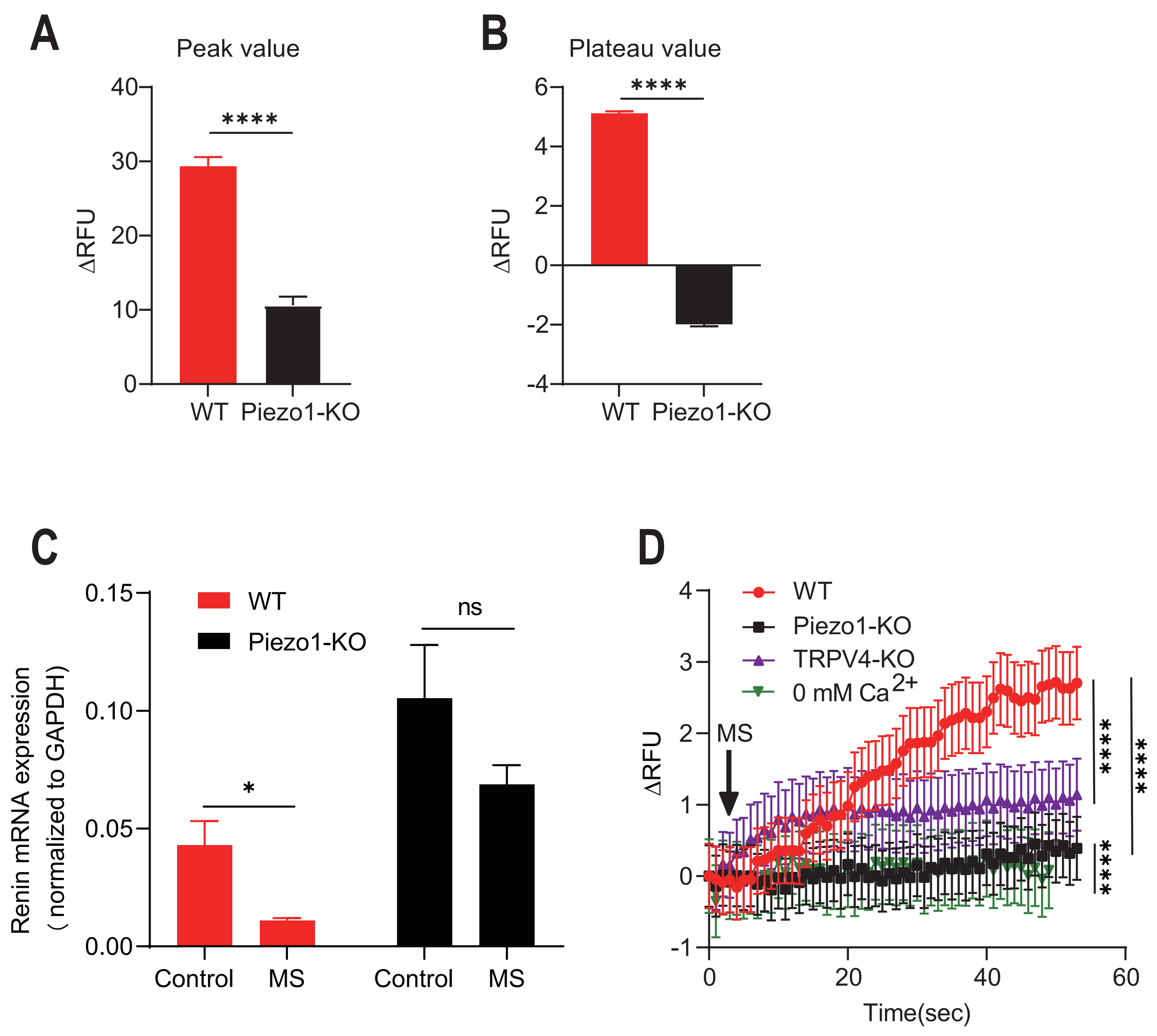
A
Peak value
ΔRFU
40
30
20
10
0
****
WT
Piezo1-KO
B
Plateau value
ΔRFU
6
4
2
0
-2
-4
****
WT
Piezo1-KO
C
Renin mRNA expression
(normalized to GAPDH)
0.15
0.10
0.05
0.00
WT
Piezo1-KO
ns
*
Control
MS
Control
MS
D
ΔRFU
4
3
2
1
0
-1
WT
Piezo1-KO
TRPV4-KO
0 mM Ca2+
MS
****
****
****
0
20
40
60
Time(sec)

Fig. S4

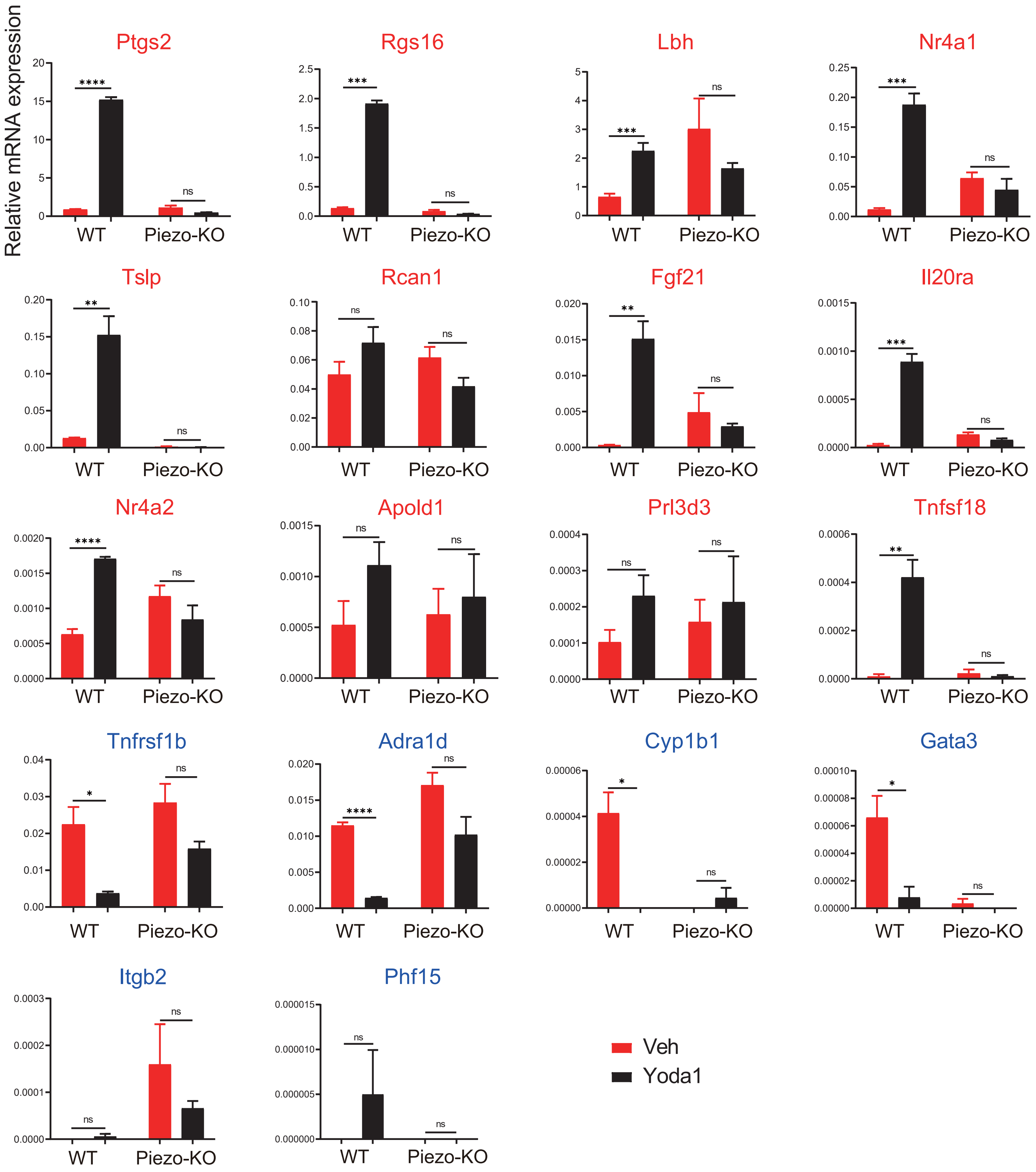
Relative mRNA expression
Ptgs2
Rgs16
Lbh
Nr4a1
Tslp
Rcan1
Fgf21
Il20ra
Nr4a2
Apold1
Prl3d3
Tnfsf18
Tnfrsf1b
Adra1d
Cyp1b1
Gata3
Itgb2
Phf15
WT
Piezo-KO
ns
*
**
***
****
Veh
Yoda1

Fig. S5

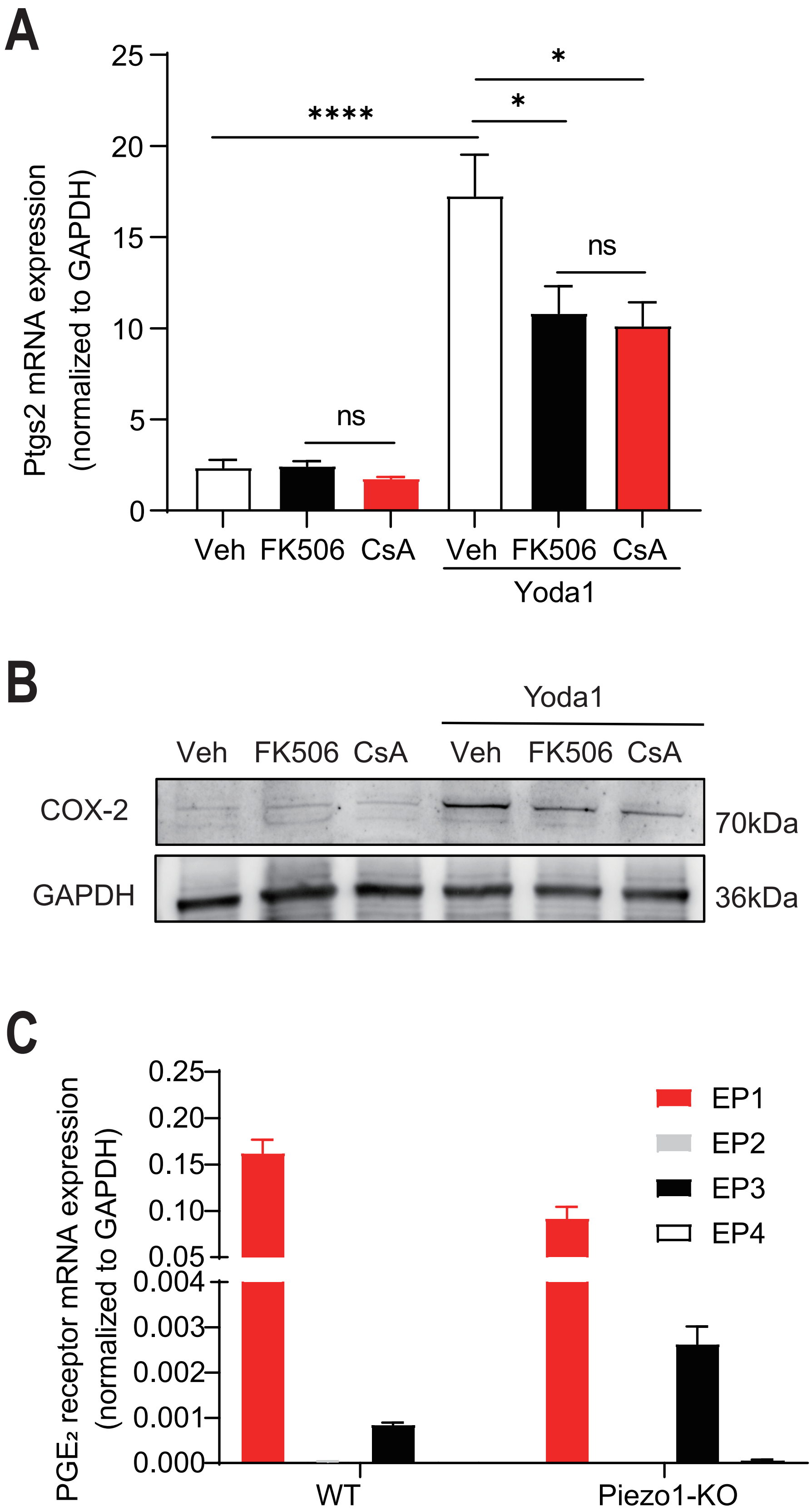
A
Ptgs2 mRNA expression
(normalized to GAPDH)
25
20
15
10
5
0
****
*
*
ns
ns
Veh FK506 CsA Veh FK506 CsA
Yoda1
B
Yoda1
Veh FK506 CsA Veh FK506 CsA
COX-2
70kDa
GAPDH
36kDa
C
PGE₂ receptor mRNA expression
(normalized to GAPDH)
0.25
0.20
0.15
0.10
0.05
0.004
0.003
0.002
0.001
0.000
EP1
EP2
EP3
EP4
WT
Piezo1-KO

Fig. S6

Uncropped blot for Figure 2C

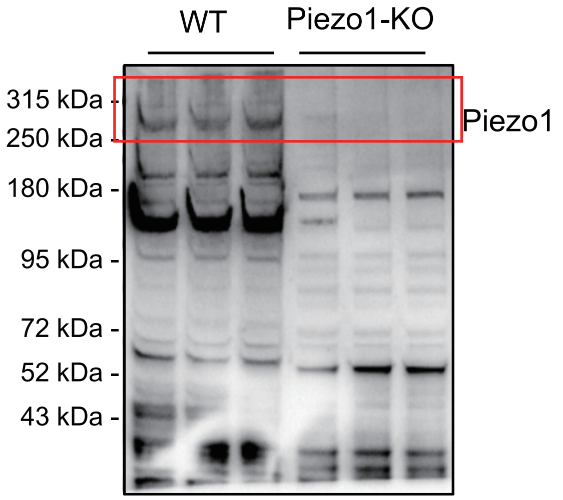

Uncropped blot for Figure 5B

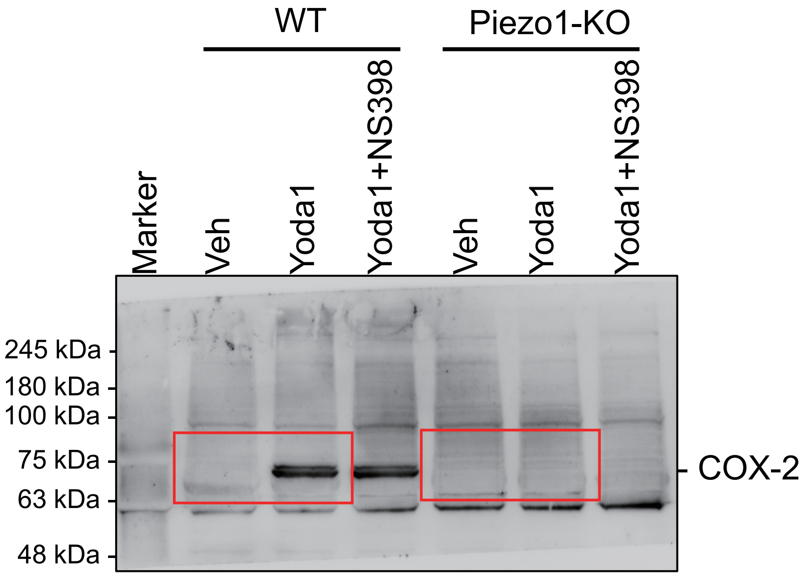

Uncropped blot for Figure S4B

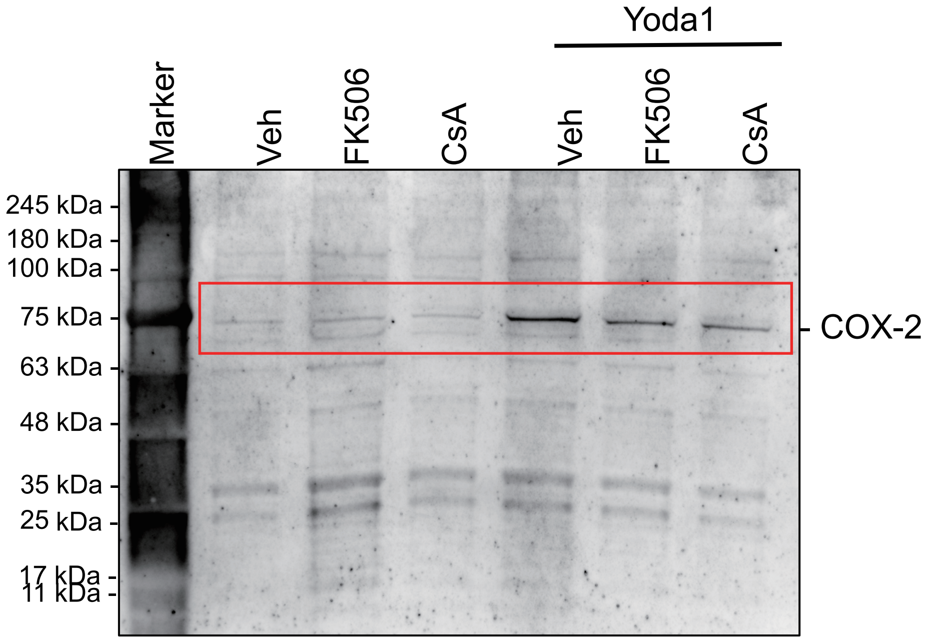

Supplement: Supplementary file 1 — Additional file 1: Fig. S1. Detection of renin in JG cells. (A) Immunofluorescence results showing the renin and αSMA expression in As4.1 cells; PMCA, a plasma membrane marker. Scale bar = 20 µm. White arrow: renin signal. (B) Immunofluorescence results showing the Piezo1 and αSMA co-expressed in mouse kidney. Scale bar = 50 µm. Green arrows: Piezo1 signals; white arrows: arterials stained by αSMA; white circles: glomerulus (G). Fig. S2. Activation of Piezo1 decreases renin expression in primary JG cells. (A) Immunofluorescence results showing the αSMA and renin co-expressed in primary mouse JG cells. Scale bar = 100 µm. (B) Calcium imaging assay showing the [Ca2+]i changes in the presence or absence of Yoda1 (20 μM) in primary mouse JG cells (n = 4). (C) Quantification of renin mRNA expression by qRT-PCR in the presence or absence of Yoda1 (20 μM) in primary mouse JG cells (n = 3). *** p < 0.001. Data are represented as mean ± SEM. Fig. S3. Mechanosensation of Piezo1-KO As4.1 cells. (A and B) Calcium imaging assay showing the Peak (A) and plateau (B) values of the [Ca2+]i responses induced by MS. (n = 3) (C) Quantification of the mRNA level in WT and Piezo1-KO As4.1 cells in response to the MS (20 dyn/cm2, n = 3). (D) Calcium imaging assay showing the [Ca2+]i changes in WT As4.1 cells in the presence or absence of extracellular Ca2+ after the MS (30 dyn/cm2, n = 3) treatment; the [Ca2+]i changes in Piezo1-KO as well as in TRPV4-KO As4.1 cells in the presence of extracellular Ca2+ after the MS (30 dyn/cm2, n = 3). ns, no significant; * p < 0.05; **** p < 0.0001. Data are represented as mean ± SEM. Fig. S4. qRT-PCR validation of DEGs in WT and Piezo1-KO As4.1 cells. DEGs from RNA-seq analysis were validated by qRT-PCR in WT and Piezo1-KO As4.1 cells with or without Yoda1 (20 µM) treatment. Red and blue colors of graph titles indicate the upregulated and downregulated genes, respectively (n = 3). ns, no significant; * p < 0.05; ** p < 0.01; *** p < 0.001; **** p < 0.0 [file 13578_2022_931_MOESM1_ESM.pdf]
