# Supplementary material for: Activation of Piezo1 downregulates renin in juxtaglomerular cells and contributes to blood pressure homeostasis
Source: Cell Biosci. 2022 Dec 5;12:197. doi: 10.1186/s13578-022-00931-2 (PMC9720979; doi:10.1186/s13578-022-00931-2)
Supplement: Supplementary file 2 — Additional file 2: Table S1. List of primers used in qRT-PCR in supplementary figures. [file 13578_2022_931_MOESM2_ESM.docx]

**Table S1. List of primers used in qRT-PCR in supplementary figures.**

| Gene | species | Forward (5’-3’) | Reverse (5’-3’) |
| --- | --- | --- | --- |
| Rcan1 | mouse | GATGGAGGAGGTGGATCTGC | ATTCAAATTTGGCCCGGCAC |
| Lbh | mouse | GATCGGCTGAGATGACCGAG | ATGGGTCCGGAAAGATCTGA |
| Ptgs2 | mouse | GCTCAGCCAGGCAGCAAATC | ACCATAGAATCCAGTCCGGGT |
| Rgs16 | mouse | CGAAGCCCCTAAAGAGGTGAA | GACTTGAGGAAGCGCGGATA |
| Tnfsf18 | mouse | ACTTCACTCAAGCCAACTGC | AGCTTCCCATCAGATGTCGT |
| Il20ra | mouse | GACAACTGGCGAGAAGTCCA | ACACTGGGACCACGTTCTTC |
| Prl3d3 | mouse | CTTTCAGGCTCCGCAGGAAT | CCTATACACATCTGCGGCCA |
| Apold1 | mouse | CCAGAGTGAAGGGGCATGG | GTTGGCCGCCAGGGAG |
| Nr4a2 | mouse | GCGCTGGGCAGGGAGAT | CCATACTGCGCCTGAACACA |
| Tslp | mouse | TCTCAGGAGCCTCTTCATCCT | ATTTGCTCGAACTTAGCCCCT |
| Fgf21 | mouse | CCTTGAAGCCAGGGGTCATT | AGGATCAAAGTGAGGCGATCC |
| Phf15 | mouse | CCTGGCTTCGGGAGCATC | GCCTCTTCTCTTCCATCTGCT |
| Tnfrsf1b | mouse | CTACAAACCGGAACCTGGGTA | TTTCACATATTGGCCAGGAGGA |
| Adra1d | mouse | TTCGTCCTGCCTCTGGGTTC | GGTAGATGAGCGGGTTCACA |
| Cyp1b1 | mouse | GGACGCCTTCATCCTCTCTG | CTGAACATCCGGGTATCTGGT |
| Itgb2 | mouse | CTGGTGCCAGAAGCTGAACT | TGCTCCTGGGGTCCATGATA |
| Gata3 | mouse | CCATTACCACCTATCCGCCC | TTCACACACTCCCTGCCTGT |
| PTGER1（EP1） | mouse | GCAGCACTGGCCCTCTTG | CATTATCGCCTGTTGGCAGC |
| PTGER 2（EP2） | mouse | GCTCCTTGCCTTTCACAATCTT | CAGGACCGGTGGCCTAAGTA |
| PTGER 3（EP3） | mouse | CGGAAGTTCTGCCAGATCAGAG | ATCTTTCCAGCTGGTCACTCC |
| PTGER 4（EP4） | mouse | TTGGAGGTAGGTCCTGAACAT | TCTAGGAATGGTACCTGTAGGG |
